# Effects of Airway Exposure to Nanoparticles on Lung Inflammation Induced by Bacterial Endotoxin in Mice

**DOI:** 10.1289/ehp.8903

**Published:** 2006-06-12

**Authors:** Ken-ichiro Inoue, Hirohisa Takano, Rie Yanagisawa, Seishiro Hirano, Miho Sakurai, Akinori Shimada, Toshikazu Yoshikawa

**Affiliations:** 1 Environmental Health Sciences Division, National Institute for Environmental Studies, Tsukuba, Japan; 2 Inflammation and Immunology, Graduate School of Medical Science, Kyoto Prefectural University of Medicine, Kyoto, Japan; 3 Department of Veterinary Pathology, Faculty of Agriculture, Tottori University, Tottori, Japan

**Keywords:** coagulatory disturbance, LPS, lung inflammation, nanoparticles

## Abstract

**Background:**

Although adverse health effects of particulate matter with a diameter of < 100 nm (nanoparticles) have been proposed, molecular and/or experimental evidence for their facilitation of lung inflammation *in vivo* is not fully defined.

**Objective:**

In the present study we investigated the effects of nanoparticles on lung inflammation related to bacterial endotoxin [lipopolysaccharide (LPS)] in mice.

**Results:**

We intratracheally administered vehicle, two sizes (14 nm, 56 nm) of carbon black nanoparticles (4 mg/kg), LPS (2.5 mg/kg), or LPS plus nanoparticles and evaluated parameters for lung inflammation and coagulation. Nanoparticles alone induced slight lung inflammation and significant pulmonary edema compared with vehicle. Fourteen-nanometer nanoparticles intensively aggravated LPS-elicited lung inflammation and pulmonary edema that was concomitant with the enhanced lung expression of interleukin-1β (IL-1β), macrophage inflammatory protein-1α (MIP-1α), macrophage chemoattractant protein-1, MIP-2, and keratinocyte chemoattractant in overall trend, whereas 56-nm nanoparticles did not show apparent effects. Immunoreactivity for 8-hydroxyguanosine, a marker for oxidative stress, was more intense in the lungs from the LPS + 14-nm nanoparticle group than in those from the LPS group. Circulatory fibrinogen levels were higher in the LPS + plus 14-nm nanoparticle group than in the LPS group.

**Conclusions:**

Taken together, evidence indicates that nanoparticles can aggravate lung inflammation related to bacterial endotoxin, which is more prominent with smaller particles. The enhancement may be mediated, at least partly, via the increased local expression of proinflammatory cytokines and via the oxidative stress. Furthermore, nanoparticles can promote coagulatory disturbance accompanied by lung inflammation.

Previous epidemiologic studies have indicated that exposure to ambient particulate matter (PM) is linked to increases in mortality and morbidity related to respiratory diseases (Abbey et al. 1999; Cohen and Pope 1995). The concentration of PM of mass median aerodynamic diameter (a density-dependent unit of measure used to describe the diameter of the particle) of ≤ 10 μm (PM_10_) is related to daily hospital admissions for asthma, acute and chronic bronchiolitis, and lower respiratory tract infections (Dockery and Pope 1994), whereas the concentration of PM ≤ 2.5 μm in mass median aerodynamic diameter (PM_2.5_) is more closely associated with both acute and chronic respiratory effects and subsequent mortality (Peters et al. 1997). Among a variety of constituents involved in PM_2.5_, diesel exhaust particles (DEPs), which are small particles with carbonaceous cores (Schuetzle 1983), are important for their apparent toxicity (Morgan et al. 1997; Pandya et al. 2002). We and others have demonstrated that DEPs have respiratory toxicity with or without predisposing factors *in vivo* (Ichinose et al. 1995, 1997; Takano et al. 1997, 2002).

To date, nanoparticles (particles < 0.1 μm in mass median aerodynamic diameter) have been postulated to affect cardiopulmonary systems (Nel 2005; Peters et al. 1997; Utell and Frampton 2000). Nanoparticles are reportedly able to penetrate deeply into the respiratory tract and have a larger surface area per unit mass than do larger particles, thus resulting in a greater inflammatory response (MacNee and Donaldson 2000; Nemmar et al. 2001). Indeed, two *in vivo* studies have demonstrated that nanoparticles have marked pulmonary toxicity compared with larger particles (Ferin et al. 1992; Li et al. 1999). Recently, we have demonstrated that carbon nanoparticles can aggravate antigen-related airway inflammation (Inoue et al. 2005a). The enhancing effects are more prominent with 14-nm nanoparticles than with larger particles (56 nm) in overall trend (Inoue et al. 2005a). On the other hand, we have previously demonstrated that DEPs (8 mg/kg; Takano et al. 2002) and DEP-derived components (4 mg/kg; Yanagisawa et al. 2003) aggravate lung inflammation related to bacterial endotoxin [lipopolysaccharide (LPS)]. However, effects of nanoparticles, in particular their size effects, on pulmonary inflammatory conditions related to bacterial endotoxin have not been fully investigated. Furthermore, nanoparticles can translocate from the lung into the circulation (Nemmar et al. 2002a, 2002b, 2003), raising the possibility that nanoparticles may facilitate not only lung inflammation but also hemostatic disturbance in the circulation.

The present study was designed to elucidate the effects of two sizes of carbon black nanoparticles (14 nm or 56 nm) on lung inflammation induced by intratracheal administration of bacterial endotoxin. We also investigated the local expression of cytokines, chemokines, and 8-hydroxyguanosine (8-OHdG) in the lung. Finally, we examined the effects of airway exposure to nanoparticles on coagulatory changes.

## Materials and Methods

### Animals

We used ICR male mice, 6 weeks of age, weighing 29–33 g (Japan Clea Co., Tokyo, Japan). This strain has been reported to be highly responsive to LPS compared with Balb/c, C3H/He, or A/J mice (Haranaka et al. 1984) in all experiments. These mice were fed a commercial diet (Japan Clea Co.) and given water *ad libitum*. Mice were housed in an animal facility that was maintained at 24–26°C with 55–75% humidity and a 12/12-hr light/dark cycle.

### Study protocol

Mice were divided into six experimental groups. The vehicle group received phosphate-buffered saline, pH 7.4 (Nissui Pharmaceutical Co., Tokyo, Japan) containing 0.05% Tween 80 (Nakalai Tesque, Kyoto, Japan). The LPS group received 2.5 mg/kg LPS (Sigma Chemical, St. Louis, MO, USA) dissolved in vehicle. Each nanoparticle group received 4 mg/kg carbon black nanoparticles (14 nm, PrinteX 90; 56 nm, PrinteX 25; Degussa, Dusseldorf, Germany) suspended in vehicle. The LPS plus nanoparticle groups received LPS (2.5 mg/kg) combined with nanoparticles (4 mg/kg) in vehicle. The surface areas of 14-nm nanoparticles and 56-nm nanoparticles are 300 m^2^/g and 45 m^2^/g, respectively (disclosed by Degussa). Nanoparticles were autoclaved at 250°C for 2 hr before use. The LPS activity, which was determined by *Limulus* amebocyte lysate assay (Seikagaku-kogyo, Tokyo, Japan), was lower than the detection limit (0.001 endotoxin units per milliliter) in the nanoparticles after treatment. The suspension was sonicated for 3 min using an ultrasonic disrupter (model UD-201; Tomy Seiko, Tokyo, Japan). In each group, vehicle, LPS, nanoparticles, or LPS plus nanoparticles were dissolved in 0.1 mL aliquots and mice were inoculated once by the intratracheal route through a polyethylene tube under anesthesia with 4% halothane (Hoechst Japan, Tokyo, Japan), as previously described (Ichinose et al. 1998; Takano et al. 1997). The animals were deeply anesthetized, studied, and then sacrificed 24 hr after intratracheal administration. The studies adhered to the U.S. National Institutes of Health guidelines for the experimental use of animals (Institute of Laboratory Animal Resources 1996). All animal studies were approved by the institutional review board of the National Institute for Environmental Studies. The animals were treated humanely and with regard for alleviation of suffering.

### Bronchoalveolar lavage

Bronchoalveolar lavage (BAL) and cell counts in BAL fluid (*n* = 7–8 in each group) were conducted as previously reported (Takano et al. 1997). In brief, the trachea was cannulated after the collection of blood. The lungs were lavaged with 1.2 mL sterile saline at 37°C instilled bilaterally by syringe. The lavaged fluid was harvested by gentle aspiration. This procedure was conducted two more times. Average volume retrieved was > 90% of the 3.6 mL that was instilled; the amounts did not differ among treatments. The fluid collections were combined and cooled to 4°C. The lavaged fluid was centrifuged at 300 × *g* for 10 min, and the total cell count was determined on a fresh fluid specimen using a hemocytometer. Differential cell counts were assessed on cytologic preparations. Slides were prepared using an Autosmear (Sakura Seiki Co., Tokyo, Japan) and were stained with Diff-Quik (International Reagents Co., Kobe, Japan). A total of 500 cells were counted under oil-immersion microscopy. After BAL procedure, the lungs were removed, snap-frozen in liquid nitrogen, and stored at −80°C for enzyme-linked immunosorbent assays (ELISAs).

### Lung water content

In another experiment, after the collection of blood, the bilateral lungs were weighed immediately after exsanguination and dried in an oven at 95°C for 48 hr. Thereafter, lung water content was estimated by calculating the ratio of lung weight [wet weight – dry weight (in milligrams)] to body weight (in grams), with eight animals per group (Ichinose et al. 1995).

### Histologic evaluation

In a separate experiment, after exsanguination, the lungs were fixed by intratracheal instillation of 10% neutral phosphate-buffered formalin at a pressure of 20 cm H_2_O for at least 72 hr. Slices 2–3 mm thick of all pulmonary lobes were embedded in paraffin. Sections (3 μm thick) were stained with hematoxylin and eosin (H&E) or were subjected to immunohistochemistry. Neutrophil infiltration was expressed as the number of neutrophils per high-power field (HPF) by counting the number of neutrophils in > 30 fields at a magnification of 100× in each slide (*n* = 5 in each group). Histologic sections were evaluated in a blind fashion.

### Measurement of cytokines and chemokines in lung tissue supernatants

Frozen lungs were subsequently homogenized with 10 mM potassium phosphate buffer (pH 7.4) containing 0.1 mM EDTA (Sigma), 0.1 mM phenyl-methanesulfonyl fluoride (Nacalai Tesque, Kyoto, Japan), 1 μM pepstatin A (Peptide Institute, Osaka, Japan), and 2 μM leupeptin (Peptide Institute) as described previously (Takano et al. 1997). The homogenates were then centrifuged at 105,000 × *g* for 1 hr. The supernatants were stored at −80°C. ELISAs for tumor necrosis factor-α (TNF-α), interleukin-1β (IL-1β; Endogen, Cambridge, MA, USA), macrophage inflammatory protein-1α (MIP-1α; R&D Systems, Minneapolis, MN, USA), MIP-2 (R&D Systems), macrophage chemoattractant protein-1 (MCP-1; R&D Systems), and keratinocyte chemoattractant (KC; R&D Systems) in lung tissue supernatants were conducted according to the manufacture’s instruction (*n* = 7–8 in each group).

### Immunohistochemistry

The production of oxidative-stress–related molecules in the lung was detected by the immunohistochemical analysis using anti–8-OHdG polyclonal antibody (Japan Institute for the Control of Aging, Shizuoka, Japan; *n* = 5 in each group) using the HistoMouse-Plus kit (Zymed Laboratories, San Francisco, CA, USA). For each lung specimen, the extent and intensity of positive staining were graded on a scale of 0–4+ by two blinded observers on two separate occasions using coded slides as previously described (Sano et al. 1992).

### Coagulation analysis

In another experiment, after deep anesthesia, the chest and abdominal walls were opened, blood was retrieved from each mouse by cardiac puncture, collected into 3.8% sodium citrate in a ratio of 10:1, and centrifuged at 2,500 × *g* for 10 min. We measured prothrombin time (PT), activated partial thromboplastin time (APTT), fibrinogen, activated protein C (APC), and activity for von Willebrand factor (vWF) (*n* = 14–16 in each group) using a commercial kit (Diagnostica Stago, Roche, Tokyo, Japan) and an STA Compact analyzer (Diagnostica Stago, Roche) as previously described (Inoue et al. 2004).

### Statistical analysis

All data were confirmed to be normally distributed by the Kolmogorov-Smirnov test. Data are reported as mean ± SE. We determined differences between groups using analysis of variance (ANOVA; StatView, version 4.0; Abacus Concepts Inc., Berkeley, CA, USA). If differences between groups were significant (*p* < 0.05) using one-way ANOVA, Bonferoni correction was used for multiple comparisons.

## Results

### Effects of nanoparticles on airway inflammation and pulmonary edema

To estimate the magnitude of airway inflammation, we examined the cellular profile of BAL fluid 24 hr after intratracheal instillation (Table 1). Nanoparticles alone increased the numbers of total cells and neutrophils compared with vehicle, but they did not reach statistical significance. LPS exposure significantly increased the numbers compared with vehicle exposure (*p* < 0.01). The numbers were significantly greater in the LPS + 14-nm nanoparticle group than in the LPS group or the 14-nm nanoparticle group (*p* < 0.01). The numbers were greater also in the LPS + 56-nm nanoparticle group than in the 56-nm nanoparticle group (*p* < 0.01). Next, to estimate pulmonary edema, we examined lung water content 24 hr after intratracheal instillation (Table 1). Airway exposure to nanoparticles or LPS significantly enhanced the pulmonary edema compared with vehicle exposure (*p* < 0.05 for 14-nm nanoparticles, *p* < 0.01 for 56-nm nanoparticles or LPS). The value was significantly greater in the LPS + 14-nm nanoparticle group than in the LPS group (*p* < 0.05) or the 14-nm nanoparticle group (*p* < 0.01). The value was also greater in the LPS + 56-nm nanoparticle group than in the 56-nm nanoparticle group (*p* < 0.01).

### Effects of nanoparticles on histologic changes in the lung

**Figure 1:**
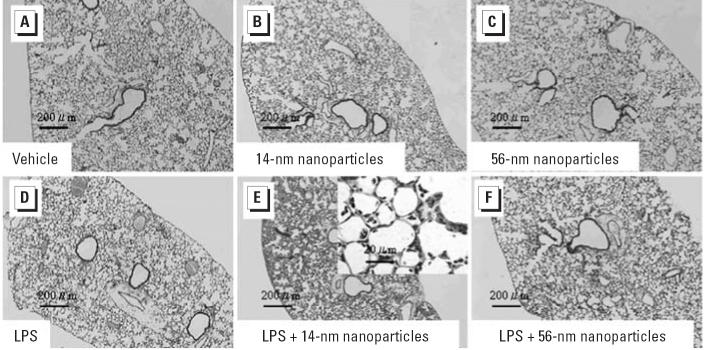
Histopathologic findings of the lung 24 hr after intratracheal administration of vehicle (*A*), two sizes of carbon black nanoparticles (14 nm, *B*; 56 nm, *C*; 4 mg/kg), LPS (2.5 mg/kg; *D*), or LPS + nanoparticles (14 nm, *E*; 56 nm, *F*). Lung histology was assessed by H&E stain; *n* = 5 per group. See “Materials and Methods” for details.

To determine the effects of nanoparticles on lung histology, we evaluated lung specimens stained with H&E 24 hr after intratracheal instillation (Figure 1). No pathologic changes were seen in the lung obtained from the vehicle group (Figure 1A). We observed slight infiltration of neutrophils in the lungs from the nanoparticle groups (Figure 1B,C) and moderate infiltration in those from the LPS group (Figure 1D). Treatment with LPS + 14-nm nanoparticles markedly enhanced neutrophil sequestration into the lung parenchyma compared with LPS alone (Figure 1E), whereas LPS + 56-nm nanoparticles did not (Figure 1F). Furthermore, we performed morphometric analysis to quantitate the number of neutrophils in the lung (Table 2). Although the number of infiltrated neutrophils in the lung parenchyma was greater in the nanoparticle groups than in the vehicle group, it did not reach significant difference. Compared with vehicle challenge, the LPS challenge significantly increased the number of neutrophils (*p* < 0.01). The LPS + 14-nm nanoparticle group showed a significantly increased number of neutrophils compared with the LPS group or the 14-nm nanoparticle group (*p* < 0.01). LPS + 56-nm nanoparticle group revealed increases in the number compared with the 56-nm nanoparticle group (*p* < 0.01) but was not significantly different from the LPS group.

### Effects of nanoparticles on the expression of proinflammatory cytokine proteins in the lung

To investigate local cytokine expression related to LPS, we measured protein levels of IL-1β and TNF-α in lung tissue supernatants 24 hr after intratracheal instillation (Table 3). LPS challenge significantly elevated the levels of IL-1β compared with vehicle challenge (*p* < 0.01). The level was significantly greater in the LPS + 14-nm nanoparticle group than in the LPS or the 14-nm nanoparticle group (*p* < 0.01). The level was greater in the LPS + 56-nm nanoparticle group than in the 56-nm nanoparticle group (*p* < 0.01). The level of TNF-α was not significantly altered among the experimental groups.

### Effects of nanoparticles on the expression of chemokine proteins in the lung

To explore local chemokine expression related to LPS, we measured protein levels of MIP-1α, MIP-2, MCP-1, and KC in lung tissue supernatants 24 hr after intratracheal instillation (Table 4). Challenge with 14-nm nanoparticles alone elevated the levels of all these chemokines compared with vehicle challenge but without significance except for KC (*p* < 0.05). LPS exposure significantly increased the levels of all the chemokines compared with vehicle exposure (*p* < 0.01). The levels were significantly greater in the LPS + 14-nm nanoparticle group than in the LPS group or the 14-nm nanoparticle group (*p* < 0.01). The level was significantly greater in the LPS + 56-nm nanoparticle group than in the 56-nm nanoparticle group (*p* < 0.01).

### Effects of nanoparticles on formations of 8-OHdG in the lung

**Figure 2:**
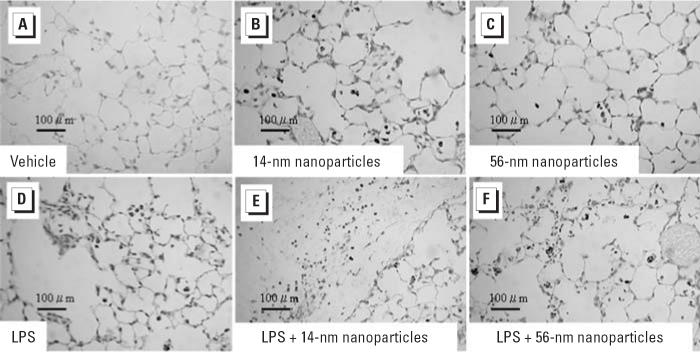
Immunohistologic staining for 8-OHdG in the lung 24 hr after intratracheal administration of vehicle (*A*), two sizes of carbon black nanoparticles (14 nm, *B*; 56 nm, *C*; 4 mg/kg), LPS (2.5 mg/kg; *D*), or LPS + nanoparticles (14 nm, *E*; 56 nm, *F*); *n* = 5 per group. See “Materials and Methods” for details.

To investigate the contribution of oxidative stress, we next studied 8-OHdG formation in the lung specimens by immunohistochemistry 24 hr after intratracheal instillation (Figure 2). In the vehicle group, positive staining for 8-OHdG was barely detectable (Figure 2A). Nanoparticle challenge induced moderate staining for 8-OHdG (Figure 2B,C). LPS challenge also induced moderate staining (Figure 2D). On the other hand, LPS plus nanoparticles resulted in intensive expression of immunoreactive 8-OHdG compared with LPS or nanoparticles alone (Figure 2E,F). The extent and intensity of the expression were strongest in the LPS + 14-nm nanoparticle group. We performed morphometric analysis to quantitate the extent and intensity of immunoreactive 8-OHdG among the experimental groups (Table 2). Compared with vehicle treatment, nanoparticle or LPS treatment revealed increased immunoreactivity for -OHdG (not significant). The immunohistochemical score with extent and intensity was greater in the nanoparticle groups than in the LPS group, although it did not show significance. However, the score was greater in the LPS plus nanoparticle groups than in the vehicle group (*p* < 0.01 for the LPS + 14-nm nanoparticle group, *p* < 0.05 for the LPS + 56-nm nanoparticle group) or the LPS group (*p* < 0.05 for the LPS + 14-nm nanoparticle group, not significant for the LPS + 56-nm nanoparticle group).

### Effects of nanoparticles on coagulatory changes

To investigate the impact of airway exposure to nanoparticles on the coagulatory system, we analyzed coagulatory parameters 24 hr after intratracheal challenge (Table 5). PT was not significantly different among the experimental groups. LPS challenge with or without nanoparticles caused prolongation of APTT compared with vehicle challenge (*p* < 0.05). APTT was further prolonged in the LPS plus nanoparticle groups compared with the LPS group, but it did not achieve statistical significance. The fibrinogen level was significantly elevated after LPS challenge (*p* < 0.01 vs. vehicle). The level was higher in the LPS plus nanoparticle groups than in the LPS group (*p* < 0.01 for the LPS + 14-nm nanoparticle group, not significant for the LPS + 56-nm nanoparticle group) or the nanoparticle groups (*p* < 0.01). LPS significantly decreased APC compared with vehicle (*p* < 0.05). The activity was further decreased in the LPS plus nanoparticle groups compared with the LPS group (not significant) or the nanoparticle groups (*p* < 0.05). Compared with the vehicle group, LPS showed a significant increase in the level of vWF (*p* < 0.05). The level was greater in the LPS + 14-nm nanoparticle group than in the LPS group (not significant) or the 14-nm nanoparticle group (*p* < 0.01).

## Discussion

The present study has demonstrated that 14-nm carbon black nanoparticles instilled intratracheally markedly enhance neutrophilic lung inflammation with pulmonary edema related to bacterial endotoxin, and 56-nm nanoparticles show fewer effects than 14-nm nanoparticles. The enhancement is paralleled by the increased local expression levels of proinflammatory cytokine such as IL-1β and chemokines such as MIP-1α, MIP-2, MCP-1, and KC. TNF-α levels were not affected by any of the treatments. In addition, combined challenge with LPS and 14-nm nanoparticles significantly increases circulatory fibrinogen level compared with challenge with LPS alone.

Epidemiologic studies have implicated the causal correlation between atmospheric concentration of PM_2.5_ and cardiopulmonary adverse effects (Brook et al. 2004; Peters et al. 1997; Samet et al. 2000). Among constituents involved in PM_2.5_, DEPs are important for their apparent toxicity (Morgan et al. 1997; Pandya et al. 2002). In fact, we have experimentally demonstrated that DEPs enhance antigen-related airway inflammation (Takano et al. 1997) and lung inflammation related to bacterial endotoxin (Takano et al. 2002) *in vivo*. DEPs are small particles with carbonaceous cores (Schuetzle 1983). Recently, we have demonstrated that carbon nanoparticles of the same sizes as those used in the present study facilitate antigen-related airway inflammation in mice (Inoue et al. 2005a). It is noteworthy that 14-nm nanoparticles predominantly enhance allergic airway inflammation, including lung expression of cytokines and chemokines related to antigen and immunoglobuin production, compared with 56-nm nanoparticles (Inoue et al. 2005a). On the other hand, we have previously shown that intratracheal instillation with DEPs (8 mg/kg; Takano et al. 2002) or the residual carbonaceous cores of DEPs after extraction with dichloromethane (washed DEPs, 4 mg/kg; Yanagisawa et al. 2003) worsens lung inflammation related to LPS in the same protocol as the present study. In the present study, 14-nm nanoparticles markedly aggravated lung inflammation related to bacterial endotoxin, which was confirmed by the counts of infiltration of inflammatory leukocytes in the BAL fluid and by the histologic assessment. On the other hand, 56-nm nanoparticles did not significantly exacerbate the inflammation. Our present study *a*) expands the understanding of the effects of environmental particles on bacterial-endotoxin–related lung inflammation *in vivo* and *b*) indicates that smaller (14 nm) particles can predominantly exaggerate bacterial endotoxin-related lung inflammation compared with larger (56 nm) particles, as well as allergic types of inflammation.

Our results apparently indicate that 14-nm nanoparticles can aggravate LPS-related lung inflammation more than 56-nm particles when the weight of particles is equal. Based on our previous studies using DEPs (Takano et al. 2002; Yanagisawa et al. 2003), we chose a dosage of 4 μg/kg of the particles. In contrast, however, it is important to note the surface area of the nanoparticles used in this study. The surface area of particles reportedly correlates with lung inflammation (Duffin et al. 2002). In our study, the surface area of the 14-nm nanoparticles was 6.7-fold larger than that of 56-nm nanoparticles (300 m^2^/g vs. 45 m^2^/g). Alternatively, our study has demonstrated not only the size effects of nanoparticles on acute lung inflammation but also the effects of their surface area and/or the effects of their number on inflammation. Unfortunately, we could not examine the effects of the nanoparticles with the same particle number in the present study. The number of smaller nanoparticles is larger than that of larger nanoparticles per unit mass. Future inhalation studies should provide better understanding of the effects of the nanoparticles on acute lung inflammation by using uniform surface area and particle numbers.

The mechanisms underlying the enhancing effects of 14-nm nanoparticles on lung inflammation remain unexplored. Pathogenesis of acute lung inflammation reportedly involves amplified lung expression of proinflammatory cytokines such as IL-1β and TNF-α and chemokines such as IL-8, MIP-1α, and MCP-1 (Martin 1999; Puneet et al. 2005; Standiford et al. 1995). In our previous work, indeed, we have confirmed the lung expression of proinflammatory cytokines and chemokines, including IL-1β, MIP-1α, MCP-1, and KC, in the lung 24 hr after intratracheal administration of LPS, DEPs, or washed DEPs, which is concomitant with the abrogated lung injuries (Takano et al. 2002; Yanagisawa et al. 2003). In the present study, as well as in our previous studies, exaggerating effects of nanoparticles on lung inflammation should be mediated, at least in part, through the enhanced lung expression of IL-1β, MIP-1α, MCP-1, MIP-2, and KC. In the present study, TNF-α was not significantly different among the experimental groups. TNF-α reportedly reaches peaks 1 hr after LPS injection (Heremans et al. 2000); this might be due to the kinetics of the response, in which this cytokine may peak much earlier and may return to normal values within 24 hr. The LPS dose we used here and in our previous studies (Takano et al. 2002; Yanagisawa et al. 2003) is high, including a 92% neutrophilic response in the BAL fluid in the present study. Alternatively, this high dose response of maximal neutrophilic influx may be responsible for the phenomenon.

Environmental particles including DEPs cause oxidative stress, leading to aggravated tissue injury (Lim et al. 1998). Nanoparticle exposure also causes oxidative stress in the lung (Nel 2005). Enhanced formation of 8-OHdG is a marker of oxidative stress and has been reported in the lung exposed to DEPs (Arimoto et al. 1999; Sanbongi et al. 2003). Further, we have recently demonstrated its enhanced formation in the murine lung exposed to LPS (Inoue et al. 2005b). In the present study, immunoreactive 8-OHdG in the lung was greater in the LPS + nanoparticle groups than in the LPS group. These results indicate that exaggerated lung injury by nanoparticles may be mediated, in part, via the enhanced oxidative stress. Interestingly, however, the nanoparticle groups showed more intense immunoreactive 8-OHdG than did the LPS group or the vehicle group. Alternatively, airway exposure to nanoparticles may cause oxidative stress in the lung independent of LPS exposure. This notion is supported by results of our recent study in which nanoparticles enhanced 8-OHdG formation in the lung in the presence or absence of antigen (Inoue et al. 2005a).

Although the effects of nanoparticles alone were mostly nonsignificant, the data suggest that they are certainly not negligible, in particular, for pulmonary edema. In contrast, the response to 14-nm nanoparticles + LPS was almost greater than the sum of the individual responses, but that to 56-nm nanoparticles + LPS was not. Thus, these observations could be considered to reflect synergistic effects of two inflammation-inducing agents such as 14-nm nanoparticles and LPS and as additive effects such as 56-nm nanoparticles and LPS.

Nanoparticles are able to penetrate deeply into the respiratory tract and can even pass through the lung to reach systemic circulation (MacNee and Donaldson 2000; Nemmar et al. 2001). Nemmar et al. (2001) have previously demonstrated that nanoparticles can migrate into circulation. In the present study, the LPS plus nanoparticle groups, specifically the LPS + 14-nm nanoparticle group, showed significantly higher fibrinogen levels compared with the LPS group. Additionally, although statistical significance was not achieved, enhanced activity of vWF induced by LPS was further increased by its combination with 14-nm nanoparticles. These findings suggest that smaller nanoparticles can facilitate coagulatory disturbance accompanied by lung inflammation. Enhancing effects of 14-nm nanoparticles on LPS-elicited pulmonary edema can further support this concept. Interestingly, exposure to nanoparticles alone did not induce significant fibrinogen production/release or activate vWF. It might be hypothesized that endothelial-epithelial damage induced by LPS and subsequent infiltrated effector leukocytes allow large amounts of smaller nanoparticles to pass easily into circulation, resulting in synergistic effects on hemostasis, including coagulatory disturbance. On the other hand, exposure to environmental particles reportedly generates local and systemic oxidative stress, which in turn induces/enhances inflammation and blood coagulation (MacNee and Donaldson 2000). Further, Nemmar et al. (2001) have demonstrated that nanoparticles instilled intratracheally rapidly diffuse from the lung into the systemic circulation *in vivo*. Therefore, it is also possible that intratracheally instilled nanoparticles enter the circulation by themselves and contribute to high susceptibility against LPS-elicited systemic inflammation and coagulatory disturbance. Future studies are needed to confirm the penetration and to address the above-mentioned hypothesis.

In the real world PM contains endotoxins; therefore, we simultaneously inhale endotoxins and PM in ambient air. In other words, we are involuntary primed by endotoxins. In addition, for the extrapolation to the human situation, it would be of interest to know whether an already existing and earlier-induced inflammation can be exacerbated by exposure to nanoparticles. Therefore, studies elucidating the effects of nanoparticles on LPS-priming and/or LPS-infected models may also provide better understanding of PM toxicology.

Finally, it can be hypothesized that LPS molecules physically adhere to the surface of nanoparticles and thus achieve concentrations in a microenvironment that enhance their proinflammatory potency. To examine the hypothesis, we centrifuged each solution from the LPS group or the two LPS plus nanoparticle groups and measured the LPS levels in the supernatants by LPS-specific *Limulus* amebocyte lysate assay. The LPS levels were nearly equal in the two groups (conducted as three independent experiments; data not shown). Thus, it is not likely that LPS adheres to nanoparticles in the present study.

In conclusion, this study has highlighted that nanoparticles enhance lung inflammation related to bacterial endotoxin. The enhancement is mediated through the increased local expression of IL-1β and chemokines. The enhancing effects are more prominent with 14-nm nanoparticles than with 56-nm nanoparticles in overall trend. Fourteen-nanometer nanoparticles also enhance coagulatory disturbance accompanied by lung inflammation. These results suggest that nanoparticles can exacerbate lung inflammation related to bacterial endotoxin and subsequent coagulatory disturbance. The aggravating effect is larger with the smaller particles.

## Figures and Tables

**Table 1:** Total cells and neutrophils in BAL fluid and lung water content (mean ± SE) after intratracheal challenge.

	Cells (×10^4^/total BAL fluid)		
Treatment	Total cells	Neutrophils	Lung weight to body weight ratio^a^
Vehicle	61.8 ± 4.7	13.9 ± 7.2	5.19 ± 0.08
14-nm nanoparticles	180.9 ± 35.8	146.6 ± 33.6	5.95 ± 0.08*
56-nm nanoparticles	222.3 ± 21.6	132.8 ± 19.1	6.73 ± 0.33**
LPS	770.9 ± 49.7**	711.6 ± 50.3**	9.01 ± 0.16**
LPS + 14-nm nanoparticles	1236.1 ± 145.7**,##,††	1134.6 ± 135.0**,##,††	9.93 ± 0.30**,#,††
LPS + 56-nm nanoparticles	826.2 ± 90.2**,††	750.6 ± 80.8**,††	9.40 ± 0.21**,††

a (Wet weight – dry weight)/body weight.

* *p* < 0.05 versus the vehicle group.

** *p* < 0.01 versus the vehicle group.

# *p* < 0.05 versus the LPS group.

## *p* < 0.01 versus the LPS group.

†† *p* < 0.01 versus the nanoparticle group.

**Table 2:** Quantitative analysis for neutrophil sequestration into the lung and immunohistochemistry (mean ± SE) after intratracheal challenge.

Treatment	Neutrophils (cell number/HPF)	8-OHdG (immunohistochemical score)
Vehicle	3.75 ± 1.34	0.63 ± 0.13
14-nm nanoparticles	8.35 ± 1.24	1.88 ± 0.34
56-nm nanoparticles	7.30 ± 1.90	1.87 ± 0.24
LPS	67.5 ± 9.30**	1.25 ± 0.14
LPS + 14-nm nanoparticles	153.6 ± 11.2**,##,††	2.88 ± 0.47**,#
LPS + 56-nm nanoparticles	75.9 ± 12.6**,††	2.25 ± 0.32*

* *p* < 0.05 versus the vehicle group.

** *p* < 0.01 versus the vehicle group.

# *p* < 0.05 versus the LPS group.

## *p* < 0.01 versus the LPS group.

†† *p* < 0.01 versus the nanoparticle group.

**Table 3:** Protein levels of cytokines in the lung tissue supernatants (mean ± SE) after intratracheal challenge.

Treatment	IL-1β(ng/total lung supernatants)	TNF-α(pg/total lung supernatants)
Vehicle	0.3 ± 0.2	704.3 ± 48.3
14-nm nanoparticles	6.1 ± 3.0	665.0 ± 45.0
56-nm nanoparticles	3.7 ± 1.6	649.6 ± 44.8
LPS	39.4 ± 4.4 **	655.0 ± 18.4
LPS + 14-nm nanoparticles	67.7 ± 11.1 **,##,††	658.4 ± 37.3
LPS + 56-nm nanoparticles	40.4 ± 10.6 **,††	693.8 ± 38.2

** *p* < 0.01 versus the vehicle group.

## *p* < 0.01 versus the LPS group.

†† *p* < 0.01 versus the nanoparticle group.

**Table 4:** Protein levels of chemokines (pg/total lung supernatants; mean ± SE) in the lung tissue supernatants after intratracheal challenge.

Treatment	MIP-1α	MIP-2	MCP-1	KC
Vehicle	12.0 ± 8.0	28.5 ± 7.9	51.1 ± 23.4	14.7 ± 10.1
14-nm nanoparticles	303.4 ± 135.9	232.7 ± 135.3	546.8 ± 161.3	817.1 ± 268.5*
56-nm nanoparticles	171.1 ± 56.1	108.6 ± 52.2	226.1 ± 68.6	344.3 ± 179.9
LPS	1941.9 ± 213.7**	1723.3 ± 205.1**	2201.0 ± 222.6**	3507.4 ± 197.2**
LPS + 14-nm nanoparticles	4131.5 ± 758.9**,##,††	3150.2 ± 340.6**,##,††	4203.2 ± 494.5**,##,††	5847.0 ± 317.0**,##,††
LPS + 56-nm nanoparticles	2281.2 ± 642.7**,††	1554.3 ± 402.1**,††	2327.0 ± 397.4**,††	3207.7 ± 469.6**,††

* *p* < 0.05 versus the vehicle group.

** *p* < 0.01 versus the vehicle group.

## *p* < 0.01 versus the LPS group.

†† *p* < 0.01 versus the nanoparticle group.

**Table 5:** Plasma coagulatory parameters after intratracheal instillation.

Treatment	PT (sec)	APTT (sec)	Fibrinogen (mg/dL)	APC (%)	vWF (%)
Vehicle	11.0 ± 0.2	26.9 ± 0.8	361.2 ± 34.1	4.3 ± 0.1	74.2 ± 3.2
14-nm nanoparticles	11.1 ± 0.1	27.7 ± 1.2	415.9 ± 16.8	3.8 ± 0.3	77.9 ± 5.1
56-nm nanoparticles	10.7 ± 0.2	26.9 ± 0.8	383.3 ± 21.9	4.3 ± 0.2	68.2 ± 3.9
LPS	10.9 ± 0.2	30.4 ± 0.4*,†	640.5 ± 24.6**	2.7 ± 0.1*	100.5 ± 6.3*
LPS + 14-nm nanoparticles	11.1 ± 0.1	31.2 ± 0.5*,†	735.9 ± 28.9**,##,††	2.6 ± 0.2*,†	112.7 ± 1.9**,††
LPS + 56-nm nanoparticles	11.2 ± 0.1	32.2 ± 0.7*,†	689.3 ± 26.4**,††	2.3 ± 0.2*,†	85.2 ± 4.6

* *p* < 0.05 versus the vehicle group.

** *p* < 0.01 versus the vehicle group.

## *p* < 0.01 versus the LPS group.

† *p* < 0.05 versus the nanoparticle group.

†† *p* < 0.01 versus the nanoparticle group.
